# Bovine Viral Diarrhea Virus in Cattle From Mexico: Current Status

**DOI:** 10.3389/fvets.2021.673577

**Published:** 2021-08-13

**Authors:** Ninnet Gomez-Romero, Julia F. Ridpath, Francisco Javier Basurto-Alcantara, Antonio Verdugo-Rodriguez

**Affiliations:** ^1^Vaccinology Laboratory, Department of Microbiology and Immunology, Facultad de Medicina Veterinaria y Zootecnia-Universidad Nacional Autónoma de México, Mexico City, Mexico; ^2^Ridpath Consulting, LLC and Ruminant Diseases and Immunology Research Unit, National Animal Disease Center, Ames, IA, United States; ^3^Molecular Microbiology Laboratory, Department of Microbiology and Immunology, Facultad de Medicina Veterinaria y Zootecnia-Universidad Nacional Autónoma de México, Mexico City, Mexico

**Keywords:** bovine viral diarrhea virus, pestivirus, genotypes, subgenotypes, Mexico

## Abstract

Bovine viral diarrhea (BVD) is an infectious disease, globally-distributed, caused by bovine Pestiviruses, endemic of cattle and other ruminant populations. BVD leads to significant economic losses to the cattle industry due to the wide range of clinical manifestations, including respiratory and gastrointestinal diseases and reproductive disorders. Within the *Pestivirus* genus of the family *Flaviviridae three* viral species are associated with BVD; *Pestivirus A* (Bovine viral diarrhea virus 1, BVDV-1), *Pestivirus B* (Bovine viral diarrhea virus 2, BVDV-2), *and Pestivirus H* (HoBi-like pestivirus, atypical ruminant pestivirus). These species are subdivided into subgenotypes based on phylogenetic analysis. The extensive genetic diversity of BVDV has been reported for several countries, where the incidence and genetic variation are more developed in Europe than in the Americas. The first report of BVDV in Mexico was in 1975; this study revealed seropositivity of 75% in cows with a clinical history of infertility, abortions, and respiratory disease. Other studies have demonstrated the presence of antibodies against BVDV with a seroprevalence ranging from 7.4 to 100%. Recently, endemic BVDV strains affecting cattle populations started to be analyzed, providing evidence of the BVDV diversity in several states of the country, revealing that at least four subgenotypes (BVDV-1a, 1b, 1c, and 2a) are circulating in animal populations in Mexico. Little information regarding BVD epidemiological current status in Mexico is available. This review summarizes available information regarding the prevalence and genetic diversity viruses associated with BVD in cattle from Mexico.

## Introduction

Bovine viral diarrhea (BVD) affects cattle and ruminants worldwide, leading to significant economic losses (1). The viruses that cause BVD are currently divided into three species within the *Pestivirus* genus; *Pestivirus A* (Bovine viral diarrhea virus 1, BVDV-1), *Pestivirus B* (Bovine viral diarrhea 2, BVDV-2), and *Pestivirus H* (HoBi-like pestivirus, atypical ruminant pestivirus) (2). Phylogenetic analysis has led to the segregation of BVDV-1 into at least 21 subgenotypes (BVDV 1a- 1u) and BVDV-2 (BVDV 2a-2d) and HoBi-like viruses into four subgenotypes (a-d) each (3). Analysis of the entire genome is still the most reliable criteria when BVDV genetic characterization is performed; although, sequences of the 5'UTR (untranslated region), E2 glycoprotein, and 3'UTR are used to assign species and subgenotypes, obtaining similar results (4–6). Additionally, these viruses may exist as two different biotypes, cytopathic (CP) and non-cytopathic (NCP), according to their activity in cell culture. Cytopathology *in vitro* is not related to pathogenicity *in vivo*. NCP biotype predominate in nature, while CP strains are rare and mostly associated with outbreaks of a rare fatal form of BVD named mucosal disease (7).

The term BVD includes a complex range of clinical presentations. In general, BVD is characterized by clinical manifestations including respiratory, gastrointestinal disorders, and reproductive failures such as congenital malformations, abortions, mummification, stillbirth, and as a result of transplacental infection, the birth of immunotolerant persistent infected animals (PI). These PI animals shed virus throughout their lifetime, play an essential role in BVD pathogenesis and represents one of the main sources of viral infection (8). Infection with viruses associated with BVD is suggested as an initiating event for the development of bovine respiratory disease complex (BRDC) (9) and also leads to an increased susceptibility to other diseases due to either immunosuppression or synergism with other viral and bacterial pathogens (10, 11). Infection in pregnant sheep, goat, pigs, and wild ruminants results in a clinical presentation similar to that seen in cattle and contact among these animal species facilitates viral transfer among domestic and non-domestic ruminants (12–15).

## Distribution of BVDV Subgenotypes

Phylogenetic approaches have been used to determine the prevalence of BVD associated species and subgenotypes within those species in different geographic locations. These studies revealed that BVDV-1 has a broader distribution than BVDV-2 and HoBi-like viruses. BVDV-1 displayed a higher genetic diversity suggested by the number of subgenotypes reported overall; the BVDV-1b has been the predominant subgenotype worldwide, followed by BVDV-1a and 1c. Regarding BVDV-2, subgenotype 2a is the most prevalent globally, whereas BVDV-2b, 2c, and 2d have been only detected in European and Asian countries (3). In addition, to date, HoBi-like viruses have only been detected in South America, Europe, and Asia but not in North America (16–18). Studies based on viruses found in Mexican cattle revealed the presence of at least four BVDV subgenotypes (BVDV 1a, 1b,1c, and BVDV 2a) with no evidence of HoBi-like viruses detected (19). Seroprevalence studies indicate an BVDV exposure since 1975 to date (20). No PI prevalence studies have been performed to date.

Characterization of BVDV subgenotypes continues to be a relevant matter of discussion due to the implications that variations have for detection, diagnosis, and vaccine efficacy. Variations among subgenotypes have demonstrated a direct impact in BVDV detection and vaccination, the latter, reported by previous studies where protection conferred against vaccines including BVDV 1a and 2a strains, does not protect against BVDV 1b strain (21). Additionally, antigenic variations between BVDV 1a and 1c have shown to be similar to that seen among BVDV 1a and 1b strain, which had also been proven (22). Viral diversity is an important feature to consider when designing diagnostic tools, efficient surveillance protocols, and vaccines for BVD control programs. Phylogenetic analysis is a useful tool for identifying the endemic subgenotypes in a population, dissemination to other regions, and emerging or reintroducing new BVDV variants.

## BVDV in Mexico

The beef and dairy cattle industries comprise the major avenues of animal-derived protein production in Mexico, representing 43% of the total livestock production^1^ These national industries include 35 million animals^2^ According to the Ministry of environment and natural resources (SEMARNAT), in Mexico, there are over 1.1 million livestock production units such as stables, farms, ranches, dairies, and feedlots with a wide heterogeneity in herds size, management, and social-economic situation. The states with the largest number of units are Veracruz, Chiapas, Oaxaca, and Guerrero (23, 24). Production systems vary from traditional backyard farms to highly specialized, high-input systems with cattle management classified as extensive, semi extensive, and intensive (25, 26). Beef and dairy farms are distributed throughout the national territory. In 2019, the entities with a higher population of animals destined for beef production were Veracruz, Jalisco, Chiapas, Chihuahua, and Michoacan; while Jalisco, Durango, Chihuahua, Coahuila, and Guanajuato were described as the states that concentrate the major animal inventory for dairies at national level (27). The annual beef and milk production reported is about 2 million t^3^ and around 12 275 million L, respectively (28). Mexico is ranked among the 10 major producers in worldwide bovine meat and milk (29, 30).

Official information and scientific reports regarding BVD in Mexico are limited; hence, the present review attempts to describe and discuss an overview of BVD current situation in Mexico using available data. BVD is considered an endemic disease with a nationwide distribution listed by the Secretary of Agriculture and Rural Development (SADER). The stance of the Mexican government is that “BVD infections are of minor risk since they can be controlled by good livestock practices and monthly mandatory notifications to the National System of Epidemiological Surveillance (SIVE)” (31). Because BVD is categorized as a non-regulated disease in Mexico no control or eradication programs have been implemented. Control and prevention activities are no mandatory; therefore, non-official or partial control programs are based only in voluntary procedures. Systematic vaccine application is considered an essential prevention tool; however, no vaccination coverage is known hitherto. Biosafety measures and monitoring are applied depending on the BVD knowledge that cattle producers and handlers have. Hence, prevention and control strategies are diverse among farms. In addition, due to underreporting, the assessment of BVD status of individuals animals or herds has not been possible. SIVE data of BVDV of 2015, 2016, 2017, 2018, 2020 and a preliminary till may, 2021 is available. A total of 5,705 cases were reported (868, 1,182, 944, 868, 1,118, and 283 cases, respectively) in bovines from several states, with no description in wildlife. Annual reports to the World Organization Animal Health (OIE) describes the National Center of Diagnosis Services in Animal Health (CENAPA) as a reference laboratory in charge of BVD diagnosis. Assays implemented for BVDV detection include viral isolation, qRT-PCR, ELISA, and viral neutralization; description of surveillance, monitoring, and border precaution as prevention and control actions are also described (32). Nevertheless, although prevention and mitigation activities are applied, there are insufficient official reports, and incomplete information regarding BVDV subgenotypes and biotypes distribution; accordingly, national BVDV genetic diversity remains unknown. Declaring BVD as a disease of slight risk could be associated with the scant information available regarding BVD incidence and the prevalence of persistently infected animals in beef and dairy herds. To date, there is no national or regional estimation of economic losses related to BVD, no surveys that establish a national BVD epidemiological status, and no available reports of BVDV vaccination coverage in cattle.

Conversely, the neighboring countries, the United States and Canada, have implemented testing strategies for BVDV diagnosis, genetic characterization, seroprevalence, and PI detection; therefore, BVDV epidemiology is better understood (33–35). Further, significant losses due to BVDV infection in beef and dairy cattle have been reported (36, 37). Implemented voluntary control activities are applied (33); however, no mandatory systematic BVD prevention, control, and eradication programs have been implemented in these countries (33, 38).

Because there is no requirement to report BVD cases in Mexico and no centralized national clearing house for diagnostic and epidemiological data, BVD is probably underdiagnosed in Mexico. The typing of viral variant and their distribution in specific geographic regions, incidence, and prevalence are yet to be determined. Moreover, no financial and economic surveys are conducted currently; consequently, no comparisons of the economic impact due to BVDV infections and benefits from the control activities are reported.

National BVD prevention and control have depended primarily on vaccination with no national coordination, using modified live (MLV) and inactivated virus vaccines licensed for Mexican cattle producers. BVDV vaccines are formulated with reference strains from the US (NADL, Singer, Oregon C24V, 296c, NY-1, and New York-93), including both biotypes with diverse combinations of these, together with other bovine viruses like *Bovine parainfluenza-3 virus, Bovine herpesvirus type 1* and *Bovine respiratory syncytial virus*; however, no vaccination register information is officially recorded.

In most cases, the purpose of vaccination is to prevent reproductive failure, gastrointestinal disorders, and respiratory disease; therefore, it is common to include BVDV1 and sometimes BVDV2 in multi-component vaccines, i.e., vaccines against BRDC. When applied improperly, unwanted effects need to be considered and evaluated; i.e., the use of MLV's of CP strains during pregnancy had led to reproductive disorders, recombination with field strains, and development of mucosal disease in PI animals (39–41). These events are rarely identified and reported in the Mexican cattle industry. Moreover, BVDV strains used in vaccines have not been tested for efficacy against Mexican field isolates; thus, further studies regarding the protection conferred in livestock will need to be done.

### BVDV Seroprevalence and Genetic Diversity

Initially, the evaluation of BVDV infections in Mexico was performed by detecting antibodies against BVDV in several herds (Table 1). Thus, the first study of BVD in Mexico was based on detecting BVDV neutralizing antibodies in 47 non-vaccinated animals with a clinical history of abortions, infertility, and respiratory signs reported seropositivity of 75% (20).

**Table 1:** Seroprevalence studies performed in cattle from Mexico.

**Number of samples**	**Animal specie analized**	**Cattle purpose**	**Vacciantion status**	**Mean BVDV Seroprevalence (%)**	**Region/state**	**BVDV Seroprevalence (%) per region**	**BVDV positive herds/total herds**	**References**
905	Bovine	Dairy	ND	70.5	Hidalgo	73.4	ND/225	(42)
					Morelos	56.5		
		Beef		62.5	Veracruz	75.4	ND/227	
					North Sonora	60.9		
					South Sonora	71.6		
					Durango	64.5		
					Baja Califoronia	52.7		
					Yucatan	60.8		
					Guerrero	63.3		
					San Luis Potosi	57.5		
					Jalisco	62.5		
					Coahuila	60.9		
					Chihuahua	61.6		
630	Bovine	Beef	Non-vaccinated	14	Yucatan	14	24/40	(43)
267	Bovine	Beef	ND	12.27	Campeche	12.27	4/4	(44)
99	Bovine	Dairy	Vaccinated	70	Queretaro	70	1/1	(45)
521	Deer	Wildlife	Non-vaccinated	63.53	Hidalgo, Coahuila	53.98	15/15	(15)
					Guerrero, Coahuila	61.03		
					Anahuac, Nuevo Leon	86.6		
					Nuevo Laredo, Tamaulipas	57.3		
					Guerrero, Tamaulipas	81.6		
3,529	Bovine	Dairy, beef and dual	Vaccinated and Non-Vaccinated	60.35	North Veracruz	64.2	ND/320	(46)
					Center Veracruz	57.5		
					South Veracruz	59.3		
417	Bovine	Dairy	Non-vaccinated	67.4	Hidalgo	81	6/6	(47)
			Vaccinated		Queretaro	100		
					Morelos	100		
		Dual			Veracruz	100		
					Tamaulipas	100		
		Beef			Chihuahua	7.4		
2,940	Bovine	ND	Non-vaccinated	81.27	San Rafael/Veracruz	80.02	7/7	(48)
					San Rafael/Veracruz	93.27		
					San Rafael/Veracruz	83.37		
					Cotaxtla/Veracruz	96.4		
					Cotaxtla/Veracruz	39.25		
					Medellin/Veracruz	80.16		
					Medellin/Veracruz	96.44		
4,487	Bovine	Dairy	Vaccinated	78.8	Aguascalientes	73	182/182	(49)
					Chiapas	83		
					Chihuahua	81		
					Guanajuato	74		
					Hidalgo	71		
					Jalisco	67		
					Laguna	71		
					Queretaro	73		
					Sinaloa	57		
			Non-vaccinated		Veracruz	74		
178	Bovine	Dairy	Non-vaccinated	46.6	Tlalmanalco/Mexico state	40	29/29	(50)
					Amecameca/Mexico State	58.2		
					Ayapango/México State	59.8		
500	Bovine	Dairy	Vaccinated	48.6	Hidalgo (Stable 1)	42	10/10	(51)
					Hidalgo (Stable 2)	52		
					Hidalgo (Stable 3)	44		
					Hidalgo (Stable 4)	54		
					Hidalgo (Stable 5)	46		
					Hidalgo (Stable 6)	58		
					Hidalgo (Stable 7)	50		
					Hidalgo (Stable 8)	46		
					Hidalgo (Stable 9)	40		
					Hidalgo (Stable 10)	54		
385	Bovine	Dairy, beef	Non-vaccinated	47.8	Matamoros/Tamaulipas	10	7/7	(52)
					Mante/Tamaulipas	42.3		
					Victoria/Tamaulipas	50.75		
					Gonzalez/Tamaulipas	41.51		
					Abasolo/Tamaulipas	62.5		
					San Fernando/Tamaulipas	31.25		
					Laredo/Tamaulipas	60.71		
421	Bovine	ND	Non-vaccinated	31.4–51.4	Ayotoxco de Guerrero/Puebla	15–25^*^	11/11	(53)
					Hueytamalco/Puebla	27.6–39.5^*^		
					Nauzontla/Puebla	15.4–38.5^*^		
					San Juan Acateno/Puebla	55.5–79.4^*^		
					Xochitlan/Puebla	57.1-71.4^*^		
				49.7–54.6	Cunduacan/Tabasco	33.3–66.7^*^	7/7	
					Huimanguillo/Tabasco	54.8–58^*^		
					Rancheria el Puente/Tabasco	41.7–58.3^*^		
				76–76.2	Cotaxtla/Veracruz	49.3–64.1^*^	6/6	
					San Rafael/Veracruz	74.4–88^*^		
					Medellin/Veracruz	82.1–85.9^*^		

*ND: No data*.

* *Values represent a two-times period of BVDV seroprevalence evaluation from the same animal population*.

Subsequently, a study by Suzan et al., described a 70.5% seroprevalence in dairy cattle from two states and 62.5% in beef cattle from 10 states (42) as serologic evidence of BVDV infection in healthy cattle. Similar results were achieved in assays of vaccinated animals belonging to farms with a record of reproductive failures associated with coinfections with pathogens like *Leptospira, Brucella, Neospora*, and infectious bovine rhinotracheitis virus (IBR). Even though vaccination can play a role in the prevalence detected, the antibody titer significantly increased in animals that have aborted should be considered (45, 49).

Previous studies in unvaccinated animals calculate a seroprevalence among 74–81.27% (47, 48, 53). Hence, the elevated level of seropositivity in these surveys indicates recent infection or the presence of a PI animal among the population surveyed (54). Further, moderate exposure levels demonstrated in evaluations in healthy animals (seropositivity of 47.8–54.6%) reveal an old BVDV exposition or an early acute infection (50, 52, 53). Similarly, studies in non-vaccinated animals with a record of abortion and miscarriage showed a seroprevalence ranging from 46.6 to 60.35% (46, 50). However, in these studies, the role of BVDV in reproductive disorders is not fully understood due to the detection of other reproductive pathogens involved and non-BVDV isolation or antigen detection performed in the aborted fetuses. Lower antibody response was detected in a southeast state, where the 14% seroprevalence reflected a natural exposure to BVDV (43).

In addition, contact between livestock and other domestic animal and wildlife species can explain these species' seroprevalence rates. For example, previous surveys in Mexico detected a 20% BVDV seropositivity in domestic goats (42). Moreover, in a population of white-tail deer, an average seroprevalence of 63.53% was detected. Factors involved in the high prevalence estimated in this study were cattle management, the prevalence of BVD in cattle in neighboring areas, and continuous grazing practices. Likewise, ranches with the highest antibody prevalence were those with cattle cohabitation compared to ranches with no cattle (15). Epidemiological data support that BVDV can be maintained in white-tail deer and capable of shedding BVDV consistent to PI cattle. Therefore, deer are considered an important BVDV source when sufficient contacts between PI deer and naïve cattle occur (55).

The serological results show that a substantial proportion of Mexican cattle has been exposed to BVDV, whether by natural exposure or vaccination; these are important criteria to consider for diagnosis purposes. However, after immunization, the BVDV antibodies titer detected results from the application of vaccines and did not reflect the natural, historical exposition of cattle to BVDV field strains. Thus, identification of antibody response requires an accurate assessment to avoid seroprevalence misinterpretation. Moreover, antibody response in seronegative immunotolerant animals should also be considered as PI cattle can respond only to heterologous BVDV strains other than the specific strain that induces immunotolerance (56). In addition, based on national seroprevalence studies, variations in antibody prevalence among locations within the same state and region were commonly reported. These variations may be due to differences in management practices such as addition of untested cattle and mixing of cattle from different sources in large herds (57, 58).

Little information is available regarding the genetic diversity of BVDV in cattle populations from Mexico. A recent study examining viruses found in cattle from six Mexican states detected four subgenotypes: BVDV-1a, 1b, 1c, BVDV-2a, and no evidence of HoBi-like viruses were reported (19). In this study, BVDV-1c was the most frequently detected subgenotype followed by 1b, 1a, and 2a, representing a unique prevalence pattern of BVDV subgenotypes reported in North America. In comparison, BVDV-1a, 1b, and 2a are the subgenotypes predominantly detected in cattle from the US and Canada, while BVDV-1c subgenotype has not been detected (22). In addition, BVDV subgenotype 1b was detected in healthy water buffaloes and isolated from a captive fallow deer from Mexican wildlife (59). Moreover, the detection of the pestivirus border disease virus (BDV) genotype 1 has been described in clinically healthy cattle from Mexico (60), reinforcing the fact that close contact between animal species is a risk factors for interspecies transfer. The latter has important implications in BVD control because other ruminant pestiviruses can cause misinterpretations in BVDV tests, as many tests used to detect BVDV do not differentiate among BVDV and BDV infections (61).

Surveillance and monitoring of BVDV variants circulating in the Mexican cattle population are crucial for establishing national/regional epidemiological status and to better understand BVDV ecology in Mexico. The information yielded would contribute to the development of efficacious control strategies specific to Mexico.

## Author Contributions

NG-R: writing—original draft and writing—review & editing. FJB-A and AV-R: review & editing. JR: writing, review, conceptualization, and editing & funding acquisition. All authors contributed to the article and approved the submitted version.

## Conflict of Interest

JR was employed by Ridpath Consulting, LLC. The remaining authors declare that the research was conducted in the absence of any commercial or financial relationships that could be construed as a potential conflict of interest.

## Publisher's Note

All claims expressed in this article are solely those of the authors and do not necessarily represent those of their affiliated organizations, or those of the publisher, the editors and the reviewers. Any product that may be evaluated in this article, or claim that may be made by its manufacturer, is not guaranteed or endorsed by the publisher.

## Footnotes

^1^http://www.campomexicano.gob.mx/boletinsiap/019e.html#:~:text=El%20centro%20del%20pa%C3%ADs%20genera,para%20el%20ganado%20en%20pie (accessed May 15, 2021).

^2^https://www.gob.mx/siap/documentos/poblacion-ganadera-136762 (accessed May 15, 2021).

^3^https://www.gob.mx/agricultura/prensa/la-produccion-de-carne-de-res-en-mexico-mantiene-un-crecimiento-anual-sostenible-del-2-5-agricultura#:~:text=De%20acuerdo%20con%20el%20panorama,en%2061.6%20millones%20de%20toneladas (accessed May 15, 2021).
